# From Waste to Preservation: Assessing the Protective Effect of Fruit By-Products Extracts on the Oxidative Stability of Edible Vegetable Oils

**DOI:** 10.3390/foods15132379

**Published:** 2026-07-03

**Authors:** Henry I. Castro-Vargas, Fabián Parada-Alfonso

**Affiliations:** 1 Grupo de Investigación en Electroquímica y Medio Ambiente (GIEMA/CICBA), Departamento de Ciencias Naturales, Extractas y Estadística, Facultad de Ciencias Básicas, Universidad Santiago de Cali, Campus Pampalinda, Calle 5 No 62-00, Santiago de Cali 760035, Colombia; 2 Grupo de Investigación en Química de Alimentos (GIQA), Departamento de Química, Facultad de Ciencias, Universidad Nacional de Colombia, Carrera 30 No 45-03, Bogota 111321, Colombia; fparadaa@unal.edu.co

**Keywords:** fruit by-products, natural antioxidants, phenolic compounds, lipid oxidation, edible vegetable oils, orange peel, pineapple peel, circular bioeconomy

## Abstract

The valorization of agro-industrial fruit by-products as sources of natural antioxidants represents a sustainable strategy to replace synthetic additives in food preservation. This study systematically evaluated the phenolic composition and antioxidant activity of extracts from thirteen underutilized Colombian fruit by-products (peels, seeds, and calyxes) and assessed their protective effect against lipid oxidation in an edible vegetable oil (EVO) model system over 15 days at 60 °C. Total phenolic content (TPC) ranged from 23.1 to 2553.2 mg GAE/100 g. Orange peel (OP) and pineapple peel (PP) exhibited the highest TPC and strongest antioxidant activity, effectively inhibiting the formation of lipid hydroperoxides, hexanal, nonanal, and TBARS, and outperforming synthetic antioxidants (BHA, BHT, TBHQ) in several parameters. Multivariate analyses classified the extracts into high-efficacy, moderate-to-low efficacy, and pro-oxidant groups. HPLC-ESI-MS/MS characterization of OP and PP revealed diverse phenolic acids (gallic, caffeic, ferulic, p-coumaric, vanillic, sinapic) and flavonoids (quercetin, rutin, catechin, C-glycosylated derivatives), which are related to the antioxidant properties observed. Pearson correlation analysis revealed a positive correlation between TPC and oxidation inhibition (r = 0.89–0.94). These findings demonstrate that Colombian fruit by-products, particularly OP and PP, are promising sustainable sources of natural antioxidants for enhancing the oxidative stability of edible vegetable oils within a circular bioeconomy framework.

## 1. Introduction

Edible vegetable oils (EVOs) are a fundamental component of the human diet, primarily due to their high content of essential fatty acids (such as omega-3 and omega-6), lipophilic vitamins, and their contribution as an energy source. Beyond their basic nutritional value, certain EVOs, notably olive and avocado oils, are also recognized for containing bioactive compounds (e.g., polyphenols, tocopherols, phytosterols) that impart additional functional properties and potential health benefits [1,2]. Within the food industry, vegetable oils play a pivotal role in determining the texture, flavor, and overall stability of a wide range of food products, thereby exerting a significant influence on their shelf life and sensory attributes. Consequently, the chemical and thermal stability of these oils is a critical factor that directly affects both the nutritional value and the technological functionality of the final food products [3,4].

Despite their nutritional and technological importance, EVOs are highly susceptible to lipid oxidation, primarily due to their elevated content of polyunsaturated fatty acids (PUFAs). This oxidative process is promoted and accelerated by various factors encountered during food processing and storage, including exposure to heat, light, pro-oxidant metals, and reactive oxygen species, which trigger complex reaction mechanisms [4,5]. The oxidation process leads to the formation of several undesirable compounds, including primary products such as hydroperoxides, and secondary metabolites like aldehydes (e.g., hexanal, nonanal, malondialdehyde, and 4-hydroxy-2-nonenal), ketones, and other volatile organic molecules such as acrolein [6]. The accumulation of these compounds is directly associated with the development of rancid off-flavors, a significant loss of nutritional value, and potential toxic effects on consumers [7]. From an industrial and economic perspective, these oxidative processes significantly compromise product stability and shorten shelf life, leading to increased product losses, higher rates of consumer returns, and elevated costs associated with reformulation, specialized packaging, or modified storage requirements.

To mitigate the deleterious effects of lipid oxidation, the food industry has conventionally employed synthetic antioxidants, including butylated hydroxyanisole (BHA), butylated hydroxytoluene (BHT), and tert-butylhydroquinone (TBHQ). These compounds are widely recognized for their high efficacy as chain-breaking antioxidants, their thermal stability, and their low cost, which render them a viable and attractive technological tool for preserving a wide range of food products. However, in recent decades a growing concern among consumers and the scientific community about the safety and potential long-term toxic effects of these synthetic compounds [8,9]. This scenario has stimulated extensive scientific research focused on identifying alternative antioxidants, particularly those of natural origin, capable of replacing synthetic compounds while potentially offering additional functional benefits to foods [10,11]. Concurrently, a strong consumer trend toward clean-label products, preferably formulated with naturally derived ingredients, has emerged, further reinforcing the need for such alternatives.

Natural plant-based products, particularly fruits, represent promising sources of phenolic compounds with well-documented antioxidant properties [12,13]. These secondary metabolites not only play a crucial role in the plant’s defense against oxidative stress but also offer significant potential for applications in the food industry [14,15]. Specifically, they can contribute to the development of functional ingredients and the formulation of foods with enhanced oxidative stability and added value [16,17]. The incorporation of such natural antioxidants supports the replacement of synthetic counterparts while simultaneously aligning with the growing consumer demand for cleaner, more natural food labels.

Globally, agro-industrial waste ranks as the third largest contributor to environmental pollution, with an estimated annual production of 2.5 billion tons [18]. The decomposition of this biomass generates compounds with detrimental effects on ecosystems, significantly altering the physicochemical properties of air, soil, and water, thereby diminishing their quality for the development and habitat of living organisms [19]. Furthermore, the proper treatment and disposal of these residues entail substantial efforts and costs for the agro-industry, a concern that is amplified by the fact that current and forthcoming global legislation is increasingly oriented toward promoting environmentally sustainable production processes. However, it is now well recognized that agro-industrial residues, particularly those derived from the fruit processing industry, constitute a valuable source of bioactive compounds, including phenolic substances, which exhibit a broad spectrum of properties and potential applications [20]. Substantial scientific evidence has accumulated regarding the antioxidant properties of phenolic compounds present in seeds, peels, calyxes, and other by-products generated during fruit processing [21,22]. This has stimulated growing interest in valorizing this biomass—transforming it from waste into high-value-added products—for specific applications in sectors such as the food industry [23].

Colombia is a megadiverse agricultural country with significant potential for fruit production. With over 5.2 million hectares dedicated to agriculture, this sector led the national economy in 2024 [24]. Furthermore, during the most recent Food Summit of Alimentec 2024, held with the participation of the FAO, Colombia was reaffirmed as a potential global food pantry [25]. This agricultural prominence poses both opportunities and challenges for the production, diversification, and industrialization of the country’s fruit products. According to FAO statistics, Colombia produced approximately 11.6 million tons of fruit in 2024, with the most representative crops including banana, avocado, coffee, orange, pineapple, passion fruit, and blackberry [26]. Currently, Colombian fruit production is primarily destined for domestic consumption, with the agro-industry being one of the main demand sectors, consuming nearly 50% of the total production. This intensive agro-industrial activity is inevitably associated with the generation of substantial amounts of residues, which can reach up to 50% of the processed raw material. Consequently, it is estimated that nearly 2 million tons of residues were generated by the Colombian fruit agro-industry in 2024 alone [27].

In recent decades, numerous studies have explored the potential of biomasses such as seeds, peels, and pomace derived from the consumption and industrial processing of Colombian fruits as sources of antioxidant compounds [21,28,29]. Many of these investigations have focused on characterizing and quantifying phenolic antioxidants, as well as evaluating their antioxidant properties, primarily through in vitro models. However, comparatively few studies have examined their actual efficacy in attenuating lipid oxidation within complex food matrices, such as EVOs, or in direct food applications [30,31]. Furthermore, certain fruit residues generated in Colombia, such as those from lulo or najanjilla (*Solanum quitoense* Lam) and curuba or banana passion fruit (*Passiflora tripartita* Kunth), remain largely underexplored in this context. More importantly, to date, there is no systematic and comparative study that evaluates, under standardized conditions, the protective potential of a broad range of residues derived from Colombian biodiversity as sources of phenolic antioxidants for inhibiting lipid oxidation in vegetable oils.

Therefore, this study aimed to conduct a systematic and comparative evaluation of the phenolic composition and antioxidant properties of extracts obtained from a selection of underutilized fruit by-products generated by the Colombian agro-industry. Specifically, we investigated residues (seeds, peels, or calyxes) from ten highly produced fruits such as naranjilla, goldenberry, passion fruit, banana passion fruit, coffee, soursop, guava, orange, pineapple and blackberry. The antioxidant properties of these extracts were assessed by monitoring their protective effect against lipid oxidation in an EVO model system. A kinetic approach was employed to compare the performance of the by-product extracts with that of synthetic antioxidants (BHA, BHT, and TBHQ) and gallic acid as a natural antioxidant. Furthermore, multivariate statistical analyses, including principal component analysis (PCA) and cluster analysis (CA), were applied to identify patterns, similarities, and groupings among the residues based on their antioxidant properties. Finally, the most representative phenolic compounds were characterized in the extracts exhibiting the highest antioxidant effect. While the general use of fruit residues as sources of antioxidants has been widely recognized, the systematic comparison of underutilized Colombian fruit by-products in a real food matrix (EVO) has not been previously reported. Moreover, several of the residues investigated herein—such as naranjilla, banana passion fruit seeds, and goldenberry calyx—remain largely unexplored as potential sources of natural additives for the food industry. Therefore, this study provides a first step toward the identification and valorization of the most promising candidates for industrial application.

## 2. Materials and Methods

### 2.1. Fruit By-Products Samples and Pretreatment

Fruit by-product samples (peels, seeds, or calyxes) were obtained from the agro-industrial processing of ten different fruits: naranjilla (*Solanum quitoense* Lam), goldenberry (*Physalis peruviana* L.), passion fruit (*Passiflora edulis* Sims), banana passion fruit (*Passiflora tripartita* Kunth), coffee (*Coffea arabica* L.), soursop (*Annona muricata* L.), guava (*Psidium guajava* L.), orange (*Citrus sinensis* L.), pineapple (*Ananas comosus* L.), and blackberry (*Rubus glaucus* Benth.) (Table 1). These residues were supplied by the agro-food company Alimentos SAS S.A. (Bogotá, Colombia), which collected the fruits from various locations in the northern and central regions of Colombia (e.g., the Magdalena, Cundinamarca, and Tolima departments) and processed them to obtain pulp or concentrate. Random sampling of approximately 100 kg of each by-product type was conducted over a one-month period. Fresh residues were cleaned and then dried at room temperature (18–20 °C) in order to avoid degradation of thermolabile phenolic compounds. Once dried, the residues were milled using a grain mill (Corona-Universal, Bogotá, Colombia). The resulting powder was passed through sieves placed on a vibratory shaker, and the particle size fraction ranging from 0.180 to 0.850 mm (+80/−16 mesh) was retained for subsequent phenolic extraction. The homogenized samples were then placed into sealed high-density polyethylene bags and stored at 4 °C in a dark and dry environment until further use.

### 2.2. Phenolic Compounds Extraction

Phenolic compounds were exhaustively extracted from the fruit by-products using a Soxhlet apparatus. Methanol was selected as the extraction solvent due to its high efficiency in recovering phenolic substances; nonetheless, for food-grade applications, ethanol, water, or their mixtures would be more appropriate. To prevent thermal degradation of heat-labile phenolic compounds, the extraction was conducted under reduced pressure (0.30 atm), which lowered methanol’s boiling point to approximately 35 °C. A 10.0 g aliquot of each sample was extracted for 24 h under continuous solvent reflux. After completion, the solvent was evaporated under vacuum, and the resulting extracts were frozen and subsequently lyophilized in a freeze dryer (Labconco Corp., Kansas City, MO, USA). The dried extracts were kept at −20 °C until further determination of total phenolic content (TPC) and antioxidant activity (AA).

### 2.3. Determination of Total Phenolic Content (TPC)

The determination of total phenolic content (TPC) in the extracts was carried out using the Folin–Ciocalteu assay, according to the protocol reported by Sánchez et al. [29]. In short, each lyophilized extract was reconstituted in methanol to reach a final concentration between 10 and 20 mg/mL. A 20 μL aliquot of this solution was combined with 1580 μL of distilled water and 100 μL of Folin–Ciocalteu reagent. After a 7 min incubation period, 300 μL of a 20% (*w*/*v*) sodium carbonate solution was added. The resulting mixture was stirred thoroughly and then left to react for 120 min in the dark at room temperature. Absorbance readings were taken at 765 nm using a UV/Vis spectrophotometer (Thermo Scientific Evolution 600, Waltham, MA, USA). The TPC values were determined by reference to a calibration curve constructed with gallic acid standards over a concentration range of 50–500 μg/mL. Results are expressed as mg of gallic acid equivalents per 100 g of dry by-product (mg GAE/100 g). All determinations were conducted in triplicate.

### 2.4. Antioxidant Activity in Edible Vegetable Oil

The antioxidant properties of each phenolic extract were assessed by monitoring their protective effect against lipid oxidation in an EVO model system. The antioxidant activity (AA) of the extracts was compared to that of the synthetic antioxidants butylated hydroxyanisole (BHT), butylated hydroxytoluene (BHT) and tert-butylhydroquinone (TBHQ), as well as the natural antioxidant gallic acid (GA). The oxidation process for the EVO, including the experimental variables and the monitoring procedures, was conducted following the methodology previously described by Hernandez-Acosta et al. [32] with slight modifications. For the antioxidant activity (AA) assays, a refined, bleached, and deodorized EVO, free of added antioxidants, was used. This oil was supplied by Duquesa S.A. (Bogotá, Colombia); it was a commercial blend of palm, soybean, and sunflower oils. Its triglyceride (TG) composition consisted of approximately 70% unsaturated fatty acids (comprising 60% oleic acid, 35% linoleic acid, and 5% other unsaturated fatty acids) and 30% saturated fatty acids, primarily stearic acid. The EVO was obtained by industrial physical refining (degumming, neutralization, bleaching, and deodorization), which is the standard process used by the supplier for producing antioxidant-free vegetable oil blends. All oil samples were stored in amber glass bottles under a nitrogen atmosphere at 4 °C prior to use to prevent autoxidation.

#### 2.4.1. Edible Vegetable Oil Oxidation

For the oxidation experiments, 20 g portions of the antioxidant-free EVO were placed into amber flasks. A FeCl_2_ solution was then added as a pro-oxidant to reach a final Fe^2+^ concentration of 3.5 mg per kilogram of oil. Subsequently, each phenolic extract was individually incorporated into the oil samples at a concentration of 300 mg of phenolic compounds per kg of oil. The amount of crude extract required to achieve this concentration was calculated for each by-product based on its measured total phenolic content (Table 1). This amount ranged from approximately 1.0 to 1.5 g of crude extract per kg of oil, depending on the phenolic richness of the extract. Concentration was selected based on the recommendations of the Codex Alimentarius General Standard for Food Additives [33], which establishes maximum permitted levels for natural antioxidants in edible fats and oils typically within the range of 100–500 mg/kg. The mixtures were then homogenized for 2 min using a vortex mixer (WiseMix^®^, DAIHAN Scientific Co., Seoul, Republic of Korea). For comparison, separate oil samples were prepared by adding BHA, BHT, TBHQ and GA, each at the same final concentration of 300 mg/kg oil. Control samples, containing no antioxidants, were prepared in the same manner and analyzed immediately (day 0) to establish baseline values. The antioxidant-enriched oil samples were subjected to accelerated oxidation by heating in an oven at 60 ± 2 °C for 15 days under continuous stirring and aeration. The progression of lipid oxidation was monitored by quantifying specific markers formed during the process. These markers included primary oxidation products (lipid hydroperoxides, LHP) and secondary oxidation products, namely hexanal (HEX), nonanal (NON), and thiobarbituric acid reactive substances (TBARS). Measurements for all these oxidation products were taken at 3-day intervals over the 15-day oxidation period. All oxidation experiments were performed with six replicates (*n* = 6) for each phenolic extract, reference antioxidant, and the Control sample.

#### 2.4.2. Measurement of Lipid Hydroperoxides

Lipid hydroperoxide (LHP) formation was assessed through the measurement of conjugated dienes, employing the protocol referenced in [32]. In brief, a 50 mg portion of the oxidized EVO sample was dissolved in isooctane, and the absorbance of the resulting solution was recorded at 234 nm with a UV-Vis spectrophotometer (VARIAN Cary 50 Conc, Palo Alto, CA, USA). The hydroperoxide concentration was then calculated using a molar extinction coefficient of ε = 26,000 M^−1^ cm^−1^ [34]. Final values are reported as millimoles of LHP per kg of EVO (mmol LHP/kg).

#### 2.4.3. Measurement of Hexanal and Nonanal

The decomposition of lipid hydroperoxides into HEX and NON was monitored by headspace solid-phase microextraction coupled with gas chromatography (HS-SPME-GC).

##### Headspace Solid-Phase Microextraction

A 500 mg portion of the oxidized EVO sample was transferred into a 20 mL amber headspace vial, followed by the addition of a magnetic stir bar. The vial was immediately sealed with a rubber septum and an aluminum crimp cap, then placed in a water bath maintained at 50 ± 1 °C with continuous stirring at 700 rpm. After a 20 min equilibration period, a solid-phase microextraction fiber coated with divinylbenzene/carboxen/polydimethylsiloxane (DVB/CAR/PDMS, 50/30 μm) was exposed to the headspace for 30 min to adsorb volatile compounds. Following extraction, the fiber was retracted and promptly inserted into the gas chromatograph injector, where thermal desorption took place, and the analytes were analyzed by gas chromatography coupled to a flame ionization detector (GC-FID).

##### Gas Chromatography Analysis

GC-FID analysis was carried out on an Agilent Technologies 6820 GC System (Palo Alto, CA, USA). The instrument was equipped with a split/splitless injector fitted with a 0.75 mm internal diameter glass splitless liner, and a DB-5 capillary column (30 m length × 0.25 mm internal diameter, 0.1 μm film thickness; Agilent J&W Scientific, Folsom, CA, USA). The temperature program for the oven was: initial temperature of 30 °C held for 2 min, then ramped to 60 °C at 2 °C/min, and finally increased to 280 °C at 20 °C/min, with a final isothermal hold of 2 min. Helium served as the carrier gas at a constant flow rate of 1.8 mL/min. The injector and detector temperatures were set at 250 °C and 300 °C, respectively. The injector was operated in splitless mode for 2 min to guarantee complete thermal desorption of the analytes from the SPME fiber. Identification of hexanal and nonanal was achieved by comparing their retention times with those of authentic reference standards. Results are reported as area units per milligram of EVO (AU/mg).

#### 2.4.4. Measurement of Thiobarbituric Acid Reactive Substances

Thiobarbituric acid reactive substances (TBARS) were quantified following a slightly modified version of our earlier protocol [32]. In short, 100 mg of oxidized EVO were transferred into a 50 mL Falcon tube, followed by the addition of 26 mM BHT (added to inhibit further oxidation during the analysis), 26 mM thiobarbituric acid, and 0.3 M trichloroacetic acid prepared in 0.2 M HCl. The mixture was vortexed for 30 s and subsequently heated in a boiling water bath for 40 min. After cooling to ambient temperature, a 5 mL portion of the aqueous layer was withdrawn, transferred to a fresh tube, and combined with 5 mL of chloroform. The resulting mixture was vortexed for 30 s and then centrifuged at 5500 rpm for 20 min using a Hettich Zentrifugen Universal 320R centrifuge (Tuttlingen, Germany). The aqueous phase was carefully separated, and its absorbance was read at 532 nm with a UV-Vis spectrophotometer (VARIAN Cary 50 Conc, Palo Alto, CA, USA). Final values, expressed as milligrams of malondialdehyde equivalents per kilogram of EVO (mg MDA/kg), were derived from a calibration curve constructed with MDA standards over a concentration range of 36–185 nM. These MDA standards were prepared by acid hydrolysis of 1,1,3,3-tetraethoxypropane.

### 2.5. Analysis of Phenolic Compounds by HPLC-ESI-MS/MS

The extracts exhibiting the highest antioxidant activity were selected for the identification of their main phenolic compounds by high-performance liquid chromatography coupled with electrospray ionization tandem mass spectrometry (HPLC-ESI-MS/MS). The analyses were performed following the procedure previously described by Castro-Vargas et al. [30], using an Agilent Technologies 1260 chromatograph (Palo Alto, CA, USA) equipped with a binary pump (G1312B), a degasser, an autosampler, and a thermostatted column compartment. Each selected extract was dissolved in acetonitrile to a final concentration of 20 mg/mL, and an aliquot of 1 μL was injected into the system. Chromatographic separation was carried out on a Bischoff ProntoSIL 300-5-C18 column (150 mm × 4 mm, 5 μm particle size) maintained at 30 °C. The mobile phase consisted of 0.1% formic acid in water (A) and acetonitrile (B) at a constant flow rate of 0.3 mL/min. The following gradient elution program was applied: 0% B (0 min), 10% B (20 min), 50% B (28 min), 70% B (30 min), 90% B (50 min), and 0% B (55 min). The initial conditions (0% B) were then maintained for 10 min for column re-equilibration.

The chromatographic system was coupled to a Quadrupole Time-of-Flight tandem mass spectrometer (Agilent Technologies 6520 Q-TOF) equipped with an electrospray ionization (ESI) source operating in negative ion mode. The MS and MS/MS parameters were set as follows: capillary voltage, 3.5 kV; drying gas (nitrogen) flow rate, 5 L/min; drying gas temperature, 300 °C; nebulizer pressure, 30 psi; skimmer voltage, 175 V; and octopole RF peak voltage, 750 V. Mass spectra were acquired over a mass-to-charge ratio (*m*/*z*) range of 50–1000. For tandem MS (MS/MS) experiments, selected precursor ions were fragmented using nitrogen as the collision gas at a temperature of 350 °C and a collision energy of 20 eV. Data acquisition and processing were performed using the MassHunter Workstation software, version 5.1 (Agilent Technologies). Phenolic compounds were tentatively identified based on their MS and MS/MS spectral information, molecular formula, and comparison with bibliographic data.

### 2.6. Experimental Design and Statistical Analysis

A randomized complete block design was employed for the antioxidant activity experiments. The treatments consisted of the extracts obtained from each fruit by-product and the reference antioxidants (BHA, BHT, TBHQ, and GA), while the concentrations of the lipid oxidation products measured at different time points were considered as the response variables.

Given the large volume of experimental data generated from the lipid oxidation assays, principal component analysis (PCA) was applied as a multivariate statistical tool to process the data and to identify patterns, differences, and similarities among the antioxidant properties of the extracts. Furthermore, hierarchical cluster analysis (CA) was performed using Euclidean distance in order to classify the fruit by-products according to their efficacy in inhibiting lipid oxidation in the EVO model system. PCA and CA were performed using the mean values (*n* = 6) of LHP, HEX, NON, and TBARS determined for each extract, reference antioxidant, and control sample at each time point (days 0, 3, 6, 9, 12, and 15). Prior to analysis, the data were mean-centered and scaled to unit variance (auto-scaled) to ensure equal weighting of all variables. The CA was conducted using the average linkage method.

All statistical analyses were performed using Statgraphics Centurion XVIII software, version 19.7.02 (Statgraphics Technologies, Inc., The Plains, VA, USA) with a confidence level of 95%. All results are presented as mean ± standard deviation (SD). Statistical differences between mean values were established by one-way analysis of variance (ANOVA), followed by Tukey’s honestly significant difference post hoc test for multiple comparisons.

## 3. Results and Discussion

Phenolic extracts were obtained from thirteen agro-industrial by-products (seeds, peels, and calyxes) derived from ten fruits that are widely cultivated and processed in Colombia. The phenolic content of each by-product was determined, and the antioxidant capacity of the extracts was evaluated based on their ability to inhibit lipid oxidation in an edible vegetable oil (EVO) model system. The by-products were further classified according to their antioxidant performance using principal component analysis (PCA) and cluster analysis (CA). Finally, selected phenolic compounds were characterized in the extracts that exhibited the highest antioxidant activity.

### 3.1. Total Phenolic Contents

Table 1 presents the TPC values determined for the evaluated by-products. TPC ranged from 23.1 ± 0.1 mg GAE/100 g (soursop seeds, SS) to 2553.2 ± 24 mg GAE/100 g (orange peel, OP). The highest TPC was observed in OP, followed by banana passion fruit seeds (BPFS, 1723 ± 14 mg GAE/100 g), pineapple peel (PP, 1581.4 ± 10 mg GAE/100 g), passion fruit peel (PFP, 1470 ± 6 mg GAE/100 g), and passion fruit seeds (PFS, 1180 ± 7 mg GAE/100 g). These findings indicate that residues such as peels or seeds from orange, banana, passion fruit, pineapple, and passion fruit represent promising sources of phenolic compounds. In contrast, by-products derived from naranjilla, soursop, and blackberry exhibited comparatively low TPC values, suggesting a more limited potential as sources of these antioxidant constituents. The trends observed in the present study are consistent with previous literature reports. Alves de Castro et al. [35] and Nunes da Silva et al. [36] reported high TPC values in orange agro-industrial by-products generated in Brazil, with values ranging from 534 ± 30 to 1782.92 ± 4.5 mg GAE/100 g. Similarly, Hernández-Montesinos et al. [37] and Polanía et al. [38] described TPC values for PP that were even higher than those obtained in the present study, with reported ranges of 882.71 ± 2 to 2296 ± 4.5 mg GAE/100 g. Likewise, Teixeira Gomes et al. [22] provided a comprehensive review on the potential of agro-industrial residues as sources of phenolic compounds, highlighting passion fruit by-products as particularly rich sources, with TPC values reaching up to 3994 mg GAE/100 g. Conversely, the low TPC values observed for naranjilla and soursop by-products in this study are in agreement with earlier findings reported by Mejía et al. [39], who ranked these materials among the lowest in terms of phenolic compound content in a study evaluating ten tropical fruits.

The results presented in Table 1 suggest that, for a given fruit, the phenolic content is generally higher in the peel than in the seeds. This trend is particularly evident in fruits such as naranjilla, passion fruit, and soursop, where the TPC values determined in the peels were between 1.2-fold (passion fruit) and 4.3-fold (soursop) higher than those observed in the corresponding seeds. A similar pattern was observed when considering all evaluated by-products collectively, with the highest TPC values predominantly associated with peel-derived materials, including the goldenberry calyx (GBC). The higher phenolic content observed in peels compared to seeds can be attributed to the physiological roles of these in plant tissues. Phenolic compounds are primarily synthesized and accumulated in the outer layers of fruits, such as the peel, where they function as a protective barrier against environmental stressors, including ultraviolet radiation, microbial attack, and oxidative damage [40,41]. Consequently, fruit peels are typically richer in phenolic compounds than internal tissues such as seeds or pulp [22,23,28]. In contrast, seeds are specialized for reproduction and storage, and their biochemical composition is more oriented toward lipids, proteins, and carbohydrates than toward secondary metabolites such as phenolics [11,28].

From a broader perspective, the TPC values observed in the evaluated by-products are consistent with trends reported across different botanical families. Fruits belonging to the Rutaceae and Passifloraceae families are generally recognized as particularly rich sources of phenolic compounds, especially in their peels and seeds [42]. In contrast, families such as Solanaceae and Annonaceae tend to exhibit lower phenolic concentrations [39]. These differences can be attributed to variations in secondary metabolism, ecological adaptation, cultivation conditions, and the distribution of phenolic compounds within plant tissues [42].

Collectively, the results from this section highlight the marked variability in phenolic content among the evaluated Colombian fruit by-products. Peel-derived residues from orange, pineapple, and passion fruit, along with seeds from banana passion fruit, emerged as particularly rich sources of phenolic compounds, whereas by-products from naranjilla, soursop, and blackberry showed comparatively limited potential. These compositional differences are consistent with established trends at both the tissue and botanical family levels, underscoring the importance of selecting appropriate raw materials for the development of natural antioxidant ingredients for food applications.

### 3.2. Antioxidant Activity in Edible Vegetable Oil

The antioxidant properties of each phenolic extract were assessed by monitoring their protective effect against lipid oxidation in an edible vegetable oil (EVO) model system over a 15-day period. Their antioxidant activity (AA) was compared with that of the reference antioxidants BHA, BHT, TBHQ, and gallic acid (GA). Lipid oxidation was monitored through the formation of selected oxidation products, including lipid hydroperoxides (LHP), hexanal (HEX), nonanal (NON), and thiobarbituric acid reactive substances (TBARS). The results obtained are summarized in Table 2 and Table 3. The obtained results provide insight into the efficiency of the extracts and reference antioxidants in delaying the formation of oxidation products at different stages of the lipid oxidation process. LHP are primary oxidation products formed during the initial stages, whereas TBARS correspond to secondary and final oxidation products. HEX and NON are considered end-products of lipid oxidation. Accordingly, the evaluation of antioxidant properties should be based on the overall temporal trends of LHP, HEX, NON, and TBARS over the 15-day oxidation period, in order to determine whether a given extract or reference antioxidant effectively retards lipid oxidation across its different stages.

Table 2 and Table 3 show that certain extracts, including OP, PP, GBC, and coffee peel (CP), exhibited strong AA. These extracts effectively attenuated the formation of all monitored lipid oxidation products, maintaining their concentrations significantly below those observed in the control sample throughout the 15-day oxidation period. The OP extract inhibited LHP formation by more than 40% compared to the Control during the oxidation period, with this inhibition reaching up to 77% by the end of the oxidation period (day 15). A similar trend was observed for the formation of HEX, NON, and TBARS; however, in these cases, the extract reduced their production by more than 50%, with a maximum inhibition close to 60% at day 15. The PP extract, in contrast, exhibited a slightly lower antioxidant effect than OP in controlling LHP formation, although its ability to retard HEX production was comparable. Additionally, PP showed a greater capacity to reduce the formation of NON and TBARS, achieving inhibition efficiencies above 67% and 57%, respectively. The GBC and CP extracts also exhibited a notable capacity to attenuate lipid oxidation in the EVO system, although their overall efficiency was slightly lower than that observed for the OP and PP extracts. Both extracts reduced LHP formation by more than 35% by day 6 and maintained LHP concentrations below 50% of the Control levels from day 9 to day 15 of the oxidation period. Similarly, both extracts effectively controlled HEX formation, with their highest efficiency observed between days 0 and 6, reaching a maximum inhibition of 51% (CP, day 6). However, their antioxidant effect gradually decreased toward day 15. Notably, the CP extract demonstrated a strong ability to attenuate NON formation, even exceeding the performance observed for OP and PP. In contrast, its capacity to retard TBARS formation was considerably lower. On the other hand, the GBC extract exhibited a capacity to reduce TBARS formation comparable to that observed for the OP and PP extracts.

A second group of extracts—obtained from guava seeds (GS), soursop peel and seeds (SP and SS), naranjilla peel and seeds (NP and NS), and blackberry seeds (BS)—also exhibited antioxidant activity in the EVO system. These extracts delayed the formation of all monitored oxidation products throughout the 15-day study, consistently maintaining their concentrations below those observed in the Control sample. However, in contrast to the previously described extracts (OP, PP, GBC, and CP), this group showed lower efficacy in inhibiting the formation of certain secondary and final lipid oxidation products (HEX, NON, or TBARS). This behavior suggests a comparatively weaker antioxidant performance for the SP, SS, NP, NS, and BS extracts. For instance, the SP extract adequately reduced the formation of LHP, HEX, and TBARS; however, its ability to inhibit NON production was limited, reaching inhibition values below 40% by day 15. A similar trend was observed for GS, NP, NS, and BS. These extracts effectively controlled the formation of LHP and HEX, allowing, on average, only about 50% of the levels observed in the Control sample. Nevertheless, their effectiveness in delaying NON and TBARS formation was comparatively low. In this regard, EVO samples supplemented with NP, NS, and BS extracts exhibited TBARS inhibition values below 30% between days 12 and 15 of oxidation. Likewise, the SS extract demonstrated low efficiency in attenuating the formation of NON and TBARS.

Table 2 and Table 3 also present the AA results for the reference antioxidants BHT, BHA, TBHQ, and GA. Among these compounds, BHT exhibited the highest efficiency in retarding lipid oxidation in the EVO samples, followed by GA and TBHQ. In contrast, BHA showed the lowest antioxidant activity, demonstrating particularly limited effectiveness in attenuating TBARS formation. Comparing the AA of the phenolic extracts with that of the reference antioxidants revealed that the OP and PP extracts displayed superior antioxidant performance, as they more effectively attenuated the formation of all monitored oxidation products. The CP extract exhibited antioxidant activity comparable to that of BHT, whereas the GBC extract showed a performance similar to that of GA. Furthermore, extracts obtained from SP, GS, BS, NS, and NP demonstrated better antioxidant properties than BHA, although their effectiveness remained lower than that of BHT, TBHQ, and GA. These findings highlight the potential of selected Colombian fruit by-products, especially OP and PP, as viable natural alternatives to synthetic antioxidants for preserving edible vegetable oils. The variable efficacy observed among the extracts underscores the importance of phenolic composition and concentration, which will be further explored.

As previously indicated, the evaluation of the antioxidant properties of the extracts and reference compounds should be based on their efficiency in attenuating the different stages of the lipid oxidation process. In this regard, the OP and PP extracts significantly delayed the progression of the initial stage of lipid oxidation by reducing the formation of LHP. However, their most pronounced antioxidant effect was associated with the inhibition of the conversion of these primary products into secondary (e.g., TBARS) and final oxidation products (e.g., HEX and NON). This suggests that these extracts could potentially act not only as chain-breaking antioxidants but also as metal chelators, thereby interfering with the propagation phase of lipid oxidation [43]. However, further experimental studies are needed to confirm these mechanisms. A similar behavior was observed for the GBC and CP extracts, although the CP extract was slightly less effective in suppressing the transformation of LHP into TBARS. These results suggest that OP, PP, GBC, and CP constitute promising sources of phenolic extracts capable of attenuating lipid oxidation in EVO across their different stages, thereby markedly reducing the formation of compounds associated with the development of rancid off-flavors, a significant loss of nutritional value, and potential toxic effects on consumers [6,7].

To further elucidate the relationship between the phenolic composition of the fruit by-product extracts and their protective effect against lipid oxidation in the EVO system, a correlation analysis was performed between the TPC of these extracts with AA and the inhibition of the monitored oxidation markers (LHP, HEX, NON, and TBARS) at the end of the 15-day oxidation period. Extracts with higher TPC values, such as OP (2553.2 mg GAE/100 g), PP (1581.4 mg GAE/100 g), and GBC (716 mg GAE/100 g), consistently exhibited stronger antioxidant activity, as reflected by their superior capacity to reduce the formation of both primary and secondary oxidation products. In contrast, extracts with lower TPC, including NP (50.7 mg GAE/100 g), NS (33.2 mg GAE/100 g), SS (23.1 mg GAE/100 g), and BS (86.5 mg GAE/100 g), showed comparatively limited inhibition, particularly for NON and TBARS. Pearson correlation analysis revealed a positive correlation between TPC and the inhibition of LHP (r = 0.94, *p* < 0.01), HEX (r = 0.92, *p* < 0.01), NON (r = 0.91, *p* < 0.01), and TBARS (r = 0.89, *p* < 0.05). These findings indicate that the phenolic content is a key determinant of the antioxidant efficacy of the evaluated extracts in the EVO model system. However, the slightly lower correlation coefficients observed for TBARS suggest that the ability to inhibit these oxidation products may also depend on other factors, such as the specific phenolic profile and the presence of compounds with metal-chelating or hydroperoxide-decomposing activities [43]. Overall, these results support that TPC can serve as a useful preliminary indicator of antioxidant potential for fruit by-product extracts. Nevertheless, the variable efficacy observed among extracts with similar TPC values (e.g., CP vs. GBC) underscores the importance of phenolic composition and the need for direct evaluation in food systems.

On the other hand, the results revealed that extracts derived from banana passion fruit seeds (BPFS) and passion fruit peel (PFP) exerted a pro-oxidant effect on lipid oxidation in the EVO system. As shown in Table 2, the BPFS extract stimulated HEX formation throughout the entire oxidation period. Compared with the Control, EVO samples supplemented with BPFS exhibited HEX levels between 2.2-fold (day 3) and 4.6-fold (day 15) higher. Additionally, this extract also promoted NON formation between days 9 and 15 of oxidation, reaching concentrations up to 1.8-fold higher than those observed in the Control sample at day 15. A similar trend was observed for the PFP extract, which increased HEX formation by 1.3-fold (day 3) to 2.8-fold (day 15) relative to the Control. The pro-oxidant activity observed may be attributed to several factors. Although both extracts contain appreciable amounts of phenolic compounds (1723 ± 14 and 1470 ± 6 mg GAE/100 g, respectively), previous studies report that high antioxidant concentrations in extracts can promote lipid oxidation in food products [44,45]. Furthermore, certain flavonoids and phenolic acids can act as pro-oxidants in the presence of transition metal ions (e.g., Fe^2+^ or Cu^2+^) by reducing them and initiating Fenton-type reactions, thereby generating reactive oxygen species that accelerate the formation of hydroperoxides and their decomposition into aldehydes such as NON and NON [46]. Additionally, the endogenous metal content of the oil matrix itself may interact synergistically with specific phenolic constituents, shifting the balance from antioxidant to pro-oxidant behavior.

In recent decades, numerous researchers have explored the potential of natural antioxidants as protective agents against lipid oxidation in EVOs. In this context, Yurdunuseven et al. [47] and Yaragalla et al. [48] recently published comprehensive reviews on the effect of extracts and fractions obtained from plants, fruits, vegetables, and their by-products on the oxidative stability of EVOs. These reviews highlight the significant progress achieved in the search for natural antioxidants with potential applications in EVO systems; however, they also emphasize the need for further studies involving underexplored matrices, such as those addressed in the present work. To date, only a limited number of studies have reported the AA of extracts or fractions derived from the specific by-products investigated herein. For instance, Aydın et al. [49] evaluated the antioxidant properties of OP extracts in EVO under frying conditions, using a concentration of 123 ppm gallic acid equivalents. Their results demonstrated an AA comparable to that of BHT. This finding differs from the results obtained in the present study, a discrepancy that may be attributed to the lower concentration of phenolic antioxidants added to the EVO samples, as well as to the use of different oxidation conditions (accelerated storage at 60 °C versus frying conditions).

### 3.3. Principal Component Analysis and Cluster Analysis

**Figure 1:**
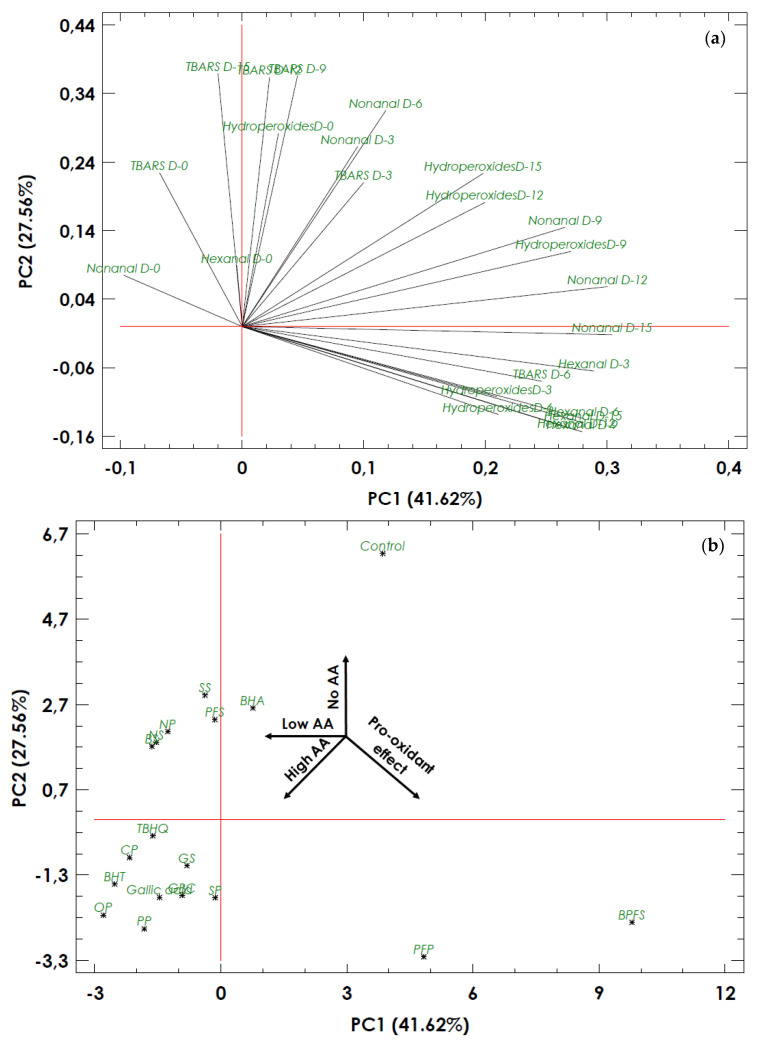
Two-dimensional plots of loadings (**a**) and scores (**b**) obtained in the principal component analysis for the antioxidant activity of fruit by-products extracts and reference antioxidants in edible vegetable oil. In the loadings plot the letters “D” and numbers correspond to the oxidation days.

To further explore the relationships among the evaluated fruit by-product extracts based on their AA in the EVO system, a principal component analysis (PCA) and a hierarchical cluster analysis (CA) were performed. These multivariate approaches allowed the identification of patterns, similarities, and groupings among the thirteen extracts and reference antioxidants, according to their capacity to inhibit the formation of the monitored oxidation products (LHP, HEX, NON, and TBARS) over the 15-day oxidation period. Figure 1 presents the loading and score plots obtained from the PCA. Two principal components (PCs) accounted for 69.48% of the total variance, with PC1 explaining the largest proportion (41.62%) and PC2 accounting for 27.56%. The selection of these components was based on their eigenvalues being greater than one (PC1: 9.98; PC2: 6.61).

According to the loadings (Figure 1a), it is possible to establish the correlations between the original variables (LHP, HEX, NON, and TBARS) and the PCs. PC1 was primarily correlated with LHP (loadings ranging from 0.19 to 0.27), HEX (0.27 to 0.28), and NON (0.10 to 0.30), with the exception of day 0 (D-0). In contrast, PC2 showed a strong correlation with TBARS (loadings between 0.21 and 0.36). These results reveal a clear distinction between the information captured by LHP, HEX, and NON, and that derived from TBARS. The correlation of PC1 with LHP, HEX, and NON indicates a close relationship among these variables. For instance, HEX is a decomposition product of linoleic acid hydroperoxides; therefore, elevated HEX levels may be associated with higher rates of LHP formation and subsequent breakdown [46]. Similarly, NON is generated from the decomposition of oleic acid hydroperoxides, following a mechanism analogous to that of HEX formation. However, the rate of NON production may vary depending on the oleic acid content and the specific oxidation conditions [50]. The association of TBARS with PC2 can be explained by the fact that these substances are secondary and final oxidation products, formed through subsequent oxidation reactions involving LHP and other intermediates, such as α,β-unsaturated aldehydes [51,52]. This separation in PCA space suggests that TBARS capture a later stage of the oxidative process, whereas LHP, HEX, and NON reflect earlier or intermediate events.

Figure 1b presents the PCA score plot, in which the AA of the extracts and reference antioxidants is represented relative to their overall mean, corresponding to the origin (X, Y = 0) of the plot. The position and distance between two points (extracts or antioxidants) are proportional to the degree of similarity or difference in their AA. For instance, OP and BPFS are located on opposite sides of PC1, indicating a marked difference in their antioxidant performance. This distinction is mainly associated with the HEX and NON levels observed in the EVO samples supplemented with these extracts. The OP extract effectively inhibited the formation of these aldehydes, whereas the BPFS extract promoted their production, exhibiting a pro-oxidant effect. In contrast, PP is positioned very close to OP along PC1, with their slight separation attributable to minor differences in the concentrations of LHP, HEX, and NON in their respective EVO samples. Accordingly, the distribution of points along PC1 provides insight into the relative ability of the extracts and antioxidants to retard the formation of LHP, HEX, and NON in EVO. Regarding PC2, a clear separation is observed between the Control and the extracts OP, PP, GBC, SP, PFP, and BPFS. This separation is explained by the significantly lower TBARS concentrations measured in these samples compared to the Control.

Considering the results discussed in Section 3.2 and the PCA scores plot (Figure 1b), a clear pattern emerges regarding the distribution of the samples. Points located in the negative regions of both PC1 and PC2 correspond to extracts and reference antioxidants with the highest capacity to inhibit lipid oxidation in EVO. This pattern is consistent with the behavior observed for OP, PP, GBC, CP, SP, BHT, and TBHQ. In contrast, points located in the positive regions of both PC1 and PC2, such as the control sample, correspond to samples lacking antioxidant activity. Additionally, points situated in the positive region of PC1 and the negative region of PC2 are associated with extracts that exhibited a pro-oxidant effect on HEX and NON formation, as observed for BPFS and PFP. Finally, a group of points located in the negative region of PC1 and the positive region of PC2 corresponds to extracts with relatively low antioxidant activity, particularly in their ability to attenuate TBARS formation.

**Figure 2:**
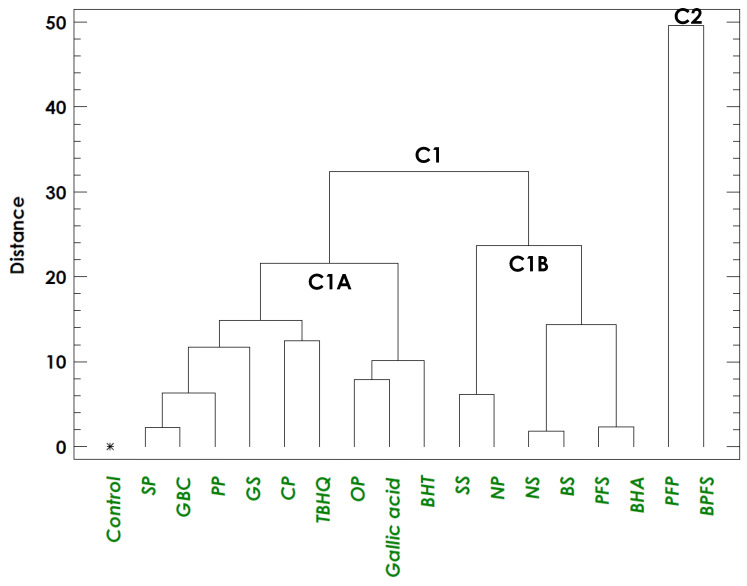
Dendrogram from the cluster analysis for the antioxidant activity of fruit by-products extracts and reference antioxidants in edible vegetable oil. * Control sample does not belong to any cluster.

Cluster analysis (CA) was performed to group the extracts and reference antioxidants according to their antioxidant properties in the EVO system. The results are presented in the dendrogram shown in Figure 2, where two independent clusters can be clearly identified: Cluster 1 (C1), comprising extracts and antioxidants with AA, and Cluster 2 (C2), consisting of extracts exhibiting a pro-oxidant effect (BPFS and PFP). Additionally, the Control sample was isolated as a distinct point, corresponding to a sample lacking AA. Cluster 1 was further subdivided into two groups, labeled C1A and C1B, which differ in the magnitude of their AA. Group C1A includes BHT, GA, OP, TBHQ, CP, GS, PP, GBC, and SP, all of which demonstrated high efficiency in delaying lipid oxidation in EVO. In contrast, group C1B comprises BHA, PFS, BS, NS, NP, and SS, which were associated with lower antioxidant capacity. Within C1A, the AA of the OP extract was classified as similar or very close to that of the reference antioxidant GA. A comparable trend was observed for the CP extract relative to TBHQ, as well as between the SP and GBC extracts. Notably, the PP extract was differentiated within C1A, which may be attributed to its greater efficiency in attenuating the different stages of lipid oxidation in EVO throughout the 15-day oxidation period.

In summary, the PCA and CA consistently classified the thirteen fruit by-product extracts and the reference antioxidants into distinct groups based on their ability to inhibit lipid oxidation in the EVO system. The joint interpretation of the score plots and the dendrogram allowed the identification of three main functional categories: (i) extracts with high antioxidant efficacy (OP, PP, GBC, CP, and SP), comparable to the synthetic antioxidants BHT and TBHQ; (ii) extracts with moderate to low antioxidant activity (GS, PFS, BS, NS, NP, and SS), which delayed primary oxidation but were less effective against TBARS formation; and (iii) extracts with a pro-oxidant effect (BPFS and PFP), which promoted the formation of hexanal and nonanal. The Control sample (without antioxidants) remained isolated, confirming the expected lack of protection. These results demonstrate that multivariate analysis is a valuable tool for discriminating the antioxidant performance of complex natural extracts, and they reinforce the potential of selected Colombian fruit by-products, particularly OP and PP, as sustainable sources of natural antioxidants for EVO preservation.

### 3.4. Characterization of Phenolic Compounds

The extracts exhibiting the highest antioxidant activity in the EVO system, namely OP and PP, were selected for the identification of their main phenolic constituents by HPLC-ESI-MS/MS. Table 4 and Table 5 summarize the retention times, molecular ions, and characteristic MS/MS fragments of the tentatively identified compounds. Eleven compounds were identified in the OP extract, including phenolic acids, glycosylated flavonoids, as well as flavanone and flavone glycosides. In contrast, seven compounds were detected in the PP extract, mainly phenolic acids and flavonoids. The fragmentation patterns of all identified compounds were compared with previously reported data in the literature and supported by evidence of their occurrence in the studied matrices [53,54,55,56,57].

The phenolic profile identified in the OP extract (Table 4) reveals a chemically diverse composition dominated by phenolic acids and flavonoids, which provides a basis for explaining its outstanding antioxidant performance in the EVO system. A first relevant feature is the presence of low-molecular-weight phenolic acids, including gallic, caffeic, ferulic, and p-coumaric acids. These compounds are widely recognized as primary antioxidants due to their ability to donate hydrogen atoms or electrons, thereby stabilizing lipid radicals and interrupting the propagation phase of lipid oxidation [58,59]. In particular, gallic acid, with its trihydroxylated structure, is hypothesized that this compound exhibit a high redox potential, which enhances its radical-scavenging capacity. Similarly, caffeic and ferulic acids, belonging to the hydroxycinnamic acid family, are known to act as both radical scavengers and metal chelators in vitro, contributing to the inhibition of Fe^2+^-catalyzed oxidation reactions in the EVO system [59]. The simultaneous presence of these compounds suggests a synergistic contribution to the inhibition of primary oxidation products (LHP), as observed in Section 3.2.

In addition, the OP extract contained several flavonoids, among which rutin and quercetin stand out due to their well-documented antioxidant properties. Quercetin is considered a potent natural antioxidant in food systems owing to its catechol structure in the B-ring and the presence of a double bond conjugated with a 4-oxo function, which could enhance electron delocalization and radical stabilization [60]. In EVO systems, quercetin inhibits the formation of lipid oxidation products by reacting with early intermediates in the lipid oxidation reactions [61]. Rutin, although glycosylated, has demonstrated high efficiency in attenuating lipid oxidation in various food systems [62].

Notably, several flavanone and flavone glycosides characteristic of citrus by-products were also identified, including naringin, neoeriocitrin, rhoifolin, and apigenin- and diosmetin-di-C-glucosides. Some of these compounds have been previously associated with the in vitro antioxidant properties of OP extracts, probably through mechanisms involving radical scavenging and Fe^2+^ chelation [53]. However, their antioxidant effect on lipid oxidation in food systems such as EVO has been poorly explored. The present study therefore provides new evidence on the protective effects of these flavanone and flavone glycosides against lipid oxidation in a real food system. Furthermore, the identification of di-C-glycosylated flavonoids (e.g., apigenin-6,8-di-C-glucoside and diosmetin-6,8-di-C-glucoside) is particularly relevant, as these compounds are considered more resistant to hydrolysis and thermal degradation than O-glycosides. This structural stability may contribute to the sustained antioxidant activity of the OP extract throughout the 15-day oxidation period.

Overall, the phenolic composition of the OP extract reflects a combination of compounds capable of acting at different stages of lipid oxidation: (i) phenolic acids that efficiently inhibit radical initiation and propagation, (ii) flavonoid aglycones with strong radical-scavenging capacity and the ability to block early intermediates, and (iii) C-glycosylated flavonoids that provide enhanced stability and prolonged activity. This compositional synergy likely underlies the superior antioxidant performance of the OP extract compared to both other by-product extracts and several reference antioxidants.

On the other hand, four phenolic acids, two flavonoids, and one glycosylated phenolic acid were identified in the PP extract (Table 5). As previously noted, gallic and p-coumaric acids are recognized for their ability to stabilize free radicals and inhibit the propagation reactions of lipid oxidation. This property has also been reported for vanillic acid in albumin–linoleic acid model systems [58]. Furthermore, the presence of quercetin in the PP extract further supports its high antioxidant activity in foods, particularly in EVO systems, and confirms its significant contribution to reducing lipid oxidation.

Catechin is a flavonoid that has been extensively studied due to its diverse properties and potential applications in food systems. Numerous studies have demonstrated the positive effects of this compound, as well as catechin-rich extracts, on the stability and shelf life of various food products. This flavonoid has shown high effectiveness in retarding lipid oxidation in a wide range of matrices, including meat systems (meat and meat products), baked goods (cakes, starch-based products, and bread), and edible oils [63]. Boroski et al. [64] reported that catechin inhibits the formation of lipid peroxides in matrices containing linseed oil under oxidative conditions. Additionally, it delays the formation of secondary oxidation products such as HEX and propanal. These findings, together with the aforementioned literature, are consistent with the results obtained in the present study for the PP extract, suggesting that catechin contributes significantly to its antioxidant activity. Specifically, catechin appears to act by directly inhibiting the formation of LHP and their subsequent conversion into secondary oxidation products such as HEX and NON.

In summary, the characterization of the phenolic profiles of the OP and PP extracts revealed distinct compositions dominated by phenolic acids and flavonoids, which correlate with their superior antioxidant performance in the EVO system. The OP extract exhibited a more diverse array of compounds, including multiple C-glycosylated flavanones and flavones, which may contribute to prolonged oxidative stability due to their resistance to thermal degradation. In contrast, the PP extract presented a simpler profile, yet the presence of catechin, along with gallic acid, quercetin, and other phenolic acids, appears to confer a potent capacity to inhibit both the formation of primary lipid hydroperoxides and their subsequent breakdown into volatile aldehydes. Collectively, these findings demonstrate that the phenolic composition is a key determinant of the protective effect observed against lipid oxidation in EVOs.

## 4. Conclusions

The present study systematically evaluated the phenolic composition and antioxidant activity of thirteen underutilized Colombian fruit by-products in an edible vegetable oil model system. This approach allows for the direct assessment of their protective effect against lipid oxidation under standardized conditions, providing practical and industry-relevant insights. Several residues proved to be effective natural antioxidants, with performance comparable to or exceeding that of synthetic antioxidants (BHA, BHT, TBHQ). Orange peel and pineapple peel exhibited a high total phenolic content and the strongest protective effect against lipid oxidation, significantly inhibiting the formation of primary (lipid hydroperoxides) and secondary (hexanal, nonanal, TBARS) oxidation products over 15 days. Multivariate analyses (PCA and CA) classified the extracts into three categories: high-efficacy antioxidants (Orange peel, pineapple peel, goldenberry calyx, coffee peel, and soursop peel), moderate-to-low efficacy antioxidants (guava seeds, passion fruit seeds, blackberry seeds, naranjilla, and soursop seeds), and pro-oxidant extracts (banana passion fruit seeds and passion fruit peel), the latter of which promoted aldehyde formation. HPLC-ESI-MS/MS characterization revealed that the superior performance of OP and PP extracts is associated with diverse phenolic acids (gallic, caffeic, ferulic, p-coumaric, vanillic, and sinapic acids) and flavonoids (quercetin, rutin, catechin, and C-glycosylated derivatives). The positive correlation between total phenolic content and oxidation inhibition (r = 0.89–0.94) supports TPC as a useful preliminary indicator, although the specific phenolic profile remains critical.

From a sustainability perspective, this study demonstrates that Colombian fruit processing residues—particularly orange and pineapple peels—can be effectively valorized as sources of natural antioxidants, contributing to waste reduction and clean-label strategies. Future research should focus on food-grade extraction processes and validation in real food systems. Overall, these findings provide a scientific basis for the sustainable use of Colombian fruit by-products to enhance the oxidative stability of edible vegetable oils within a circular bioeconomy framework.

## Figures and Tables

**Table 1:** Fruit by-products evaluated as sources of phenolic antioxidants for edible vegetable oil protection.

Common Name	Scientific Name	Family	By-Product	Abbreviation ^1^	TPC ^2^ (mg GAE/100 g)
Naranjilla	*Solanum quitoense* Lam	Solanaceae	Peel Seeds	NP NS	50.7 ± 0.4 ^k^ 33.2 ± 0.6 ^l^
Goldenberry	*Physalis peruviana* L.		Calyxes	GBC	716 ± 3 ^f^
Passion fruit	*Passiflora edulis* Sims	Passifloraceae	Peel Seeds	PFP PFS	1470 ± 6 ^d^ 1180 ± 7 ^e^
Banana passion fruit	*Passiflora tripartita* Kunth		Seeds	BPFS	1723 ± 14 ^b^
Coffee	*Coffea arabica* L.	Rubiaceae	Peel	CP	696.8 ± 1 ^g^
Soursop	*Annona muricata* L.	Anonaceae	Peel Seeds	SP SS	100.5 ± 0.5 ^i^ 23.1 ± 0.1 ^m^
Guava	*Psidium guajava* L.	Mirtaceae	Seeds	GS	223.4 ± 1 ^h^
Orange	*Citrus sinensis* L.	Rutaceae	Peel	OP	2553.2 ± 24 ^a^
Pineapple	*Ananas comosus* L.	Bromeliaceae	Peel	PP	1581.4 ± 10 ^c^
Blackberry	*Rubus glaucus* Benth	Rosaceae	Seeds	BS	86.5 ± 0.9 ^j^

^1^ Abbreviations used in the manuscript for each fruit by-product. ^2^ TPC columns show the mean ± standard deviation (*n* = 3). Means in columns followed by the same letter are not statistically different according to the ANOVA test.

**Table 2:** Lipid hydroperoxides and hexanal formed during the oxidation of edible vegetable oil samples added with fruit by-products extracts, synthetic antioxidants (BHT, BHA and TBHQ) and gallic acid.

EVO Samples	Hydroperoxides mmol LHP/kg of EVO						Hexanal AU/mg of EVO					
	Oxidation Time (Days)						Oxidation Time (Days)					
	0	3	6	9	12	15	0	3	6	9	12	15
Control	11.04 ^au^	11.28 ^ev^	22.52 ^aw^	32.22 ^ax^	52.92 ^ay^	54.69 ^az^	0.20 ^au^	0.92 ^dv^	1.47 ^dw^	2.98 ^cx^	4.07 ^cy^	4.79 ^cz^
SP	6.91 ^eu^	11.21 ^fw^	12.54 ^gv^	20.17 ^ex^	23.43 ^iz^	22.98 ^ly^	0.17 ^fu^	0.59 ^hv^	1.36 ^ew^	1.68 ^dx^	1.81 ^jy^	2.04 ^kz^
SS	11.02 ^aw^	10.21 ^hv^	9.19 ^nu^	17.34 ^hx^	19.29 ^ky^	44.40 ^bz^	0.21 ^du^	0.42 ^jv^	0.73 ^lw^	1.00 ^mx^	1.39 ^ly^	2.25 ^jz^
NP	11.03 ^aw^	9.46 ^jv^	6.64 ^qu^	13.88 ^lx^	19.29 ^ky^	22.28 ^lz^	0.22 ^cu^	0.42 ^jv^	0.61 ^mw^	0.92 ^nx^	1.39 ^ly^	1.78 ^nz^
NS	7.35 ^du^	7.64 ^nv^	7.87 ^pw^	19.29 ^fx^	27.86 ^fy^	36.46 ^ez^	0.22 ^cv^	0.22 ^mv^	0.80 ^jw^	1.14 ^lx^	1.52 ^ky^	1.98 ^lz^
PFP	6.10 ^fu^	13.95 ^bv^	22.08 ^aw^	24.80 ^bx^	31.56 ^dy^	36.14 ^ez^	0.20 ^du^	1.22 ^bv^	2.33 ^bw^	8.11 ^by^	10.62 ^by^	13.4 ^bz^
PFS	7.35 ^du^	10.64 ^gv^	11.70 ^iw^	22.73 ^dx^	25.02 ^hy^	27.15 ^hz^	0.22 ^cu^	0.84 ^ev^	1.03 ^gw^	1.19 ^kx^	1.84 ^iy^	2.23 ^jz^
CP	5.55 ^hu^	9.23 ^kv^	14.71 ^cw^	15.93 ^kx^	20.65 ^iy^	23.32 ^kz^	0.16 ^gu^	0.34 ^kv^	0.72 ^lw^	1.33 ^ix^	1.74 ^jy^	2.48 ^gz^
OP	5.06 ^iw^	6.90 ^ox^	12.18 ^hx^	17.83 ^hz^	8.90 ^ox^	12.18 ^oy^	0.24 ^au^	0.39 ^kv^	0.73 ^lw^	0.98 ^nx^	1.37 ^ly^	1.79 ^mz^
PP	6.91 ^ev^	10.79 ^hw^	10.96 ^jx^	17.91 ^hy^	21.23 ^jz^	20.96 ^mz^	0.18 ^eu^	0.51 ^iv^	0.62 ^mw^	1.16 ^lx^	1.38 ^ly^	1.79 ^mz^
BPFS	6.10 ^fv^	15.10 ^aw^	16.53 ^bx^	30.79 ^ay^	30.89 ^dy^	42.53 ^bz^	0.20 ^au^	2.05 ^av^	2.77 ^aw^	9.94 ^ax^	11.5 ^ay^	22.0 ^az^
GS	9.17 ^cu^	9.58 ^jv^	10.32 ^kw^	17.33 ^ix^	32.17 ^cy^	22.55 ^lz^	0.12 ^hv^	0.75 ^fw^	1.19 ^fx^	1.52 ^fx^	2.54 ^dy^	2.53 ^fz^
BS	7.34 ^du^	7.94 ^nv^	8.42 ^ow^	17.66 ^gx^	19.29 ^ky^	38.51 ^dz^	0.22 ^cv^	0.19 ^mv^	0.86 ^iw^	1.17 ^lx^	1.39 ^ly^	2.69 ^ez^
GBC	6.90 ^ev^	12.51 ^cw^	12.61 ^fw^	15.51 ^kx^	19.07 ^ly^	24.90 ^iz^	0.17 ^fu^	0.63 ^gv^	1.22 ^fw^	1.60 ^ex^	1.70 ^gy^	2.32 ^iz^
BHT	5.87 ^gw^	8.94 ^lx^	5.96 ^rw^	12.82 ^my^	13.37 ^nz^	8.46 ^px^	0.23 ^bu^	0.32 ^kv^	0.91 ^hw^	1.36 ^g,hx^	1.78 ^fy^	2.41 ^hz^
BHA	7.30 ^du^	11.91 ^dv^	12.81 ^fw^	23.38 ^cx^	25.60 ^gy^	34.24 ^fz^	0.22 ^cu^	1.01 ^cv^	1.19 ^fw^	1.59 ^ex^	2.13 ^ey^	2.57 ^fz^
TBHQ	5.54 ^hw^	9.78 ^ix^	10.86 ^jy^	17.89 ^gz^	18.00 ^mz^	18.18 ^nz^	0.15 ^gu^	0.29 ^lv^	0.95 ^hw^	1.25 ^jx^	1.90 ^hy^	2.43 ^hz^
Gallic acid	5.06 ^hu^	8.42 ^mv^	10.91 ^jw^	17.50 ^gx^	19.33 ^ky^	30.26 ^gz^	0.24 ^au^	0.84 ^ev^	1.06 ^gw^	1.39 ^gx^	1.89 ^hy^	3.21 ^dz^

All columns show the means (*n* = 6). Within each column, different superscript letters (a–r) indicate significant differences among treatments at the same oxidation time (*p* < 0.05). Within each row, different superscript letters (u–z) indicate significant differences over time for the same treatment (*p* < 0.05). SP: soursop peel; SS: soursop seeds; NP: naranjilla peel; NS: naranjilla seeds; PFP: passion fruit peel; PFS: passion fruit seeds; CP: coffee peel; OP: orange peel; PP: pineapple peel; BPFS: banana passion fruit seeds; GS: guava seeds; BS: blackberry seeds; GBC: golden berry calyx.

**Table 3:** Nonanal and TBARS formed during the oxidation of edible vegetable oil samples added with fruit by-products extracts, synthetic antioxidants (BHT, BHA and TBHQ) and gallic acid.

EVO Samples	Nonanal AU/mg of EVO						Thiobarbituric Acid Reactive Substances mg MDA/kg of EVO					
	Oxidation Time (Days)						Oxidation Time (Days)					
	0	3	6	9	12	15	0	3	6	9	12	15
Control	0.01 ^cu^	0.39 ^av^	0.69 ^aw^	0.81 ^bx^	0.96 ^by^	1.38 ^bz^	0.11 ^cv^	2.68 ^aw^	3.91 ^ax^	6.35 ^ay^	6.05 ^ay^	7.03 ^az^
SP	0.01 ^cu^	0.17 ^hv^	0.34 ^dw^	0.43 ^fx^	0.61 ^cy^	0.73 ^cz^	0.02 ^iu^	0.68 ^jv^	0.93 ^lw^	1.30 ^jx^	1.48 ^jy^	2.40 ^mz^
SS	0.01 ^cw^	0.28 ^ex^	0.66 ^az^	0.69 ^cz^	0.58 ^dy^	0.68 ^dz^	0.12 ^cu^	0.33 ^ov^	2.17 ^ew^	3.85 ^bx^	3.96 ^cy^	6.37 ^bz^
NP	0.01 ^cv^	0.32 ^dw^	0.53 ^bx^	0.67 ^cy^	0.58 ^dx^	0.78 ^bz^	0.10 ^dv^	0.07 ^qu^	2.49 ^dw^	3.75 ^bx^	3.96 ^cy^	5.27 ^dz^
NS	0.03 ^au^	0.15 ^iv^	0.39 ^c,ew^	0.47 ^ex^	0.56 ^dy^	0.63 ^fz^	0.38 ^au^	0.79 ^hv^	2.23 ^ew^	2.66 ^fx^	4.45 ^by^	4.71 ^ez^
PFP	0.01 ^cu^	0.11 ^kv^	0.22 ^iw^	0.66 ^cx^	0.95 ^by^	1.43 ^bz^	0.03 ^hu^	0.92 ^fv^	1.14 ^iw^	1.62 ^hx^	1.96 ^iy^	2.01 ^oz^
PFS	0.03 ^av^	0.37 ^bw^	0.47 ^cx^	0.53 ^dy^	0.54 ^dy^	0.61 ^fz^	0.37 ^au^	1.18 ^bv^	3.75 ^bx^	3.37 ^cw^	4.39 ^by^	4.91 ^dz^
CP	0.01 ^cv^	0.08 ^lw^	0.14 ^kx^	0.19 ^ly^	0.25 ^jz^	0.25 ^iz^	0.18 ^bu^	1.52 ^dv^	2.12 ^fw^	2.38 ^gx^	3.45 ^dy^	4.57 ^fz^
OP	0.02 ^bv^	0.15 ^iw^	0.21 ^ix^	0.24 ^ky^	0.33 ^hy^	0.56 ^gz^	0.04 ^fu^	0.14 ^pv^	1.30 ^gw^	1.52 ^ix^	1.88 ^iy^	2.67 ^lz^
PP	0.01 ^cu^	0.11 ^kv^	0.16 ^jw^	0.21 ^kx^	0.29 ^iy^	0.35 ^hz^	0.02 ^iu^	0.56 ^mu^	0.90 ^lw^	1.06 ^kx^	1.41 ^ky^	2.21 ^nz^
BPFS	0.01 ^cu^	0.28 ^ev^	0.48 ^cw^	1.19 ^ax^	1.45 ^ay^	2.52 ^az^	0.03 ^hu^	0.76 ^hv^	1.27 ^gw^	1.63 ^hx^	2.06 ^hy^	2.01 ^oz^
GS	0.01 ^cv^	0.15 ^iw^	0.26 ^gx^	0.27 ^jx^	0.47 ^ey^	0.55 ^gz^	0.12 ^cu^	0.69 ^kv^	1.12 ^iw^	1.23 ^jx^	2.57 ^gy^	3.37 ^iz^
BS	0.03 ^av^	0.14 ^iw^	0.35 ^dx^	0.51 ^dy^	0.58 ^dz^	0.52 ^gy^	0.38 ^au^	1.10 ^ev^	2.54 ^dw^	3.34 ^cx^	3.96 ^cy^	4.90 ^dz^
GBC	0.01 ^cu^	0.20 ^gv^	0.34 ^dx^	0.29 ^iw^	0.45 ^ey^	0.55 ^gz^	0.02 ^iu^	0.67 ^jv^	1.08 ^jw^	1.26 ^jx^	2.11 ^hy^	2.49 ^mz^
BHT	0.03 ^au^	0.25 ^fv^	0.32 ^fw^	0.39 ^g,hx^	0.46 ^ey^	0.65 ^ez^	0.11 ^du^	0.81 ^gv^	1.20 ^hw^	1.48 ^ix^	1.40 ^ky^	1.83 ^pz^
BHA	0.03 ^av^	0.34 ^cw^	0.53 ^bx^	0.60 ^cy^	0.60 ^cy^	0.79 ^bz^	0.38 ^av^	1.60 ^cw^	3.25 ^cx^	3.29 ^dx^	3.99 ^cy^	5.81 ^cz^
TBHQ	0.01 ^cu^	0.28 ^ev^	0.33 ^ew^	0.37 ^hx^	0.43 ^fy^	0.54 ^gz^	0.18 ^bu^	0.40 ^nv^	2.06 ^fw^	2.94 ^ex^	3.28 ^ey^	3.67 ^hz^
Gallic acid	0.03 ^au^	0.17 ^hv^	0.21 ^iw^	0.29 ^ix^	0.37 ^gy^	0.71 ^c,dz^	0.05 ^eu^	0.69 ^iv^	0.99 ^kw^	1.11 ^kx^	1.29 ^ly^	2.78 ^kz^

All columns show the means (*n* = 6). Within each column, different superscript letters (a–q) indicate significant differences among treatments at the same oxidation time (*p* < 0.05). Within each row, different superscript letters (u–z) indicate significant differences over time for the same treatment (*p* < 0.05). SP: soursop peel; SS: soursop seeds; NP: naranjilla peel; NS: naranjilla seeds; PFP: passion fruit peel; PFS: passion fruit seeds; CP: coffee peel; OP: orange peel; PP: pineapple peel; BPFS: banana passion fruit seeds; GS: guava seeds; BS: blackberry seeds; GBC: golden berry calyx.

**Table 4:** Phenolic compounds identified in orange peel extract by HPLC-ESI-MS/MS.

Rt (min)	Molecular Formula	[M−H]^−^ (*m*/*z*)	MS^2^ (*m*/*z*)	Tentative Identification	Reference
7.6	C7H6O5	169	125	Gallic acid	[53]
12.9	C9H8O4	179	135	Caffeic acid	[53]
17.6	C10H10O4	193	134, 117	Ferulic acid	[54]
18.2	C9H8O3	163	119, 153	p-Coumaric acid	[53]
18.9	C27H30O15	593	575, 503	Apigenin-6,8-di-C-glucoside	[54,55]
19.7	C27H30O16	609	301	Rutin	[53]
20.6	C28H32O16	623	533, 503	Diosmetin-6,8-di-C-glucoside	[54,55]
23.5	C27H32O15	595	287	Neoeriocitrin	[55]
27.8	C27H32O14	579	271, 235	Naringin	[55]
30.7	C27H30O14	577	463, 269	Rhoifolin	[55]
36.1	C15H10O7	301	271, 151	Quercetin	

**Table 5:** Phenolic compounds identified in pineapple peel extract by HPLC-ESI-MS/MS.

Rt (min)	Molecular Formula	[M−H]^−^ (*m*/*z*)	MS^2^ (*m*/*z*)	Tentative Identification	Reference
7.7	C_7_H_6_O_5_	169	125	Gallic acid	[56]
14.6	C_15_H_14_O_6_	289	245, 271	Catechin	[56,57]
14.9	C_11_H_12_O_5_	223	208, 193	Sinapic acid	
15.3	C_8_H_8_O_4_	167	152	Vanillic acid	[57]
18.1	C_9_H_8_O_3_	163	119, 153	p-Coumaric acid	[57]
26.4	C_17_H_22_O_10_	385	223, 208	Sinapoyl glucoside	
36.0	C_15_H_10_O_7_	301	271, 151	Quercetin	[56]

## Data Availability

The original contributions presented in this study are included in the article. Further inquiries can be directed to the corresponding author.

## Abbreviations

The following abbreviations are used in this manuscript:

AA	Antioxidant activity
ANOVA	Analysis of variance
BHA	Butylated hydroxyanisole
BHT	Butylated hydroxytoluene
BPFS	Banana passion fruit seeds
BS	Blackberry seeds
CA	Cluster analysis
CP	Coffee peel
DVB/CAR/PDMS	Divinylbenzene/carboxen/polydimethylsiloxane
EVOs	Edible vegetable oils
FAO	Food and Agriculture Organization of the United Nations
GA	Gallic acid
GAE	Gallic acid equivalents
GBC	Golden berry calyx
GC-FID	Gas chromatography coupled with a flame ionization detector
GS	Guava seeds
HEX	Hexanal
HPLC-ESI-MS/MS	High-performance liquid chromatography coupled with electrospray ionization tandem mass spectrometry
HS-SPME-GC	Solid-phase microextraction coupled with gas chromatography
LHP	Lipid hydroperoxides
MDA	Malondialdehyde
NON	Nonanal
NP	Naranjilla peel
NS	Naranjilla seeds
OP	Orange peel
PCA	Principal component analysis
PCs	Principal components
PFP	Passion fruit peel
PFS	Passion fruit seeds
PP	Pineapple peel
PUFAs	Polyunsaturated fatty acids
SP	Soursop peel
SS	Soursop seeds
TBARS	Thiobarbituric acid reactive substances
TBHQ	Tert-butylhydroquinone
TG	Triglyceride

## References

[1] Gonçalves M., Rodríguez-Pérez M., Calabrò A., Burgos-Ramos E., Accardi G., Silva P. (2024). A Narrative Review of Metabolomic Insights into Olive Oil’s Nutritional Value. Appl. Sci..

[2] Cervantes-Paz B., Yahia E.M. (2021). Avocado oil: Production and market demand, bioactive components, implications in health, and tendencies and potential uses. Compr. Rev. Food Sci. Food Saf..

[3] Hu Y., Cui W., Zhou H., Wang Z., Xu B. (2025). Co-oxidation behavior between lipid and protein in muscle food: A review. Food Sci. Anim. Prod..

[4] Zhao M., Liu Z., Zhang W., Xia G., Li C., Rakariyatham K., Zhou D. (2025). Advance in aldehydes derived from lipid oxidation: A review of the formation mechanism, attributable food thermal processing technology, analytical method and toxicological effect. Food Res. Int..

[5] Young W., Kim M.-J., Lee J. (2023). Approaches of lipid oxidation mechanisms in oil matrices using association colloids and analysis methods for the lipid oxidation. Food Sci. Biotechnol..

[6] Grootveld M. (2022). Evidence-Based Challenges to the Continued Recommendation and Use of Peroxidatively-Susceptible Polyunsaturated Fatty Acid-Rich Culinary Oils for High-Temperature Frying Practises: Experimental Revelations Focused on Toxic Aldehydic Lipid Oxidation Products. Front. Nutr..

[7] Sarkisyan V., Kochetkova A.A., Bessonov V., Glazkova V. (2016). Toxicological characteristics of the main lipid oxidation products. Probl. Nutr..

[8] Wang W., Xiong P., Zhang H., Zhu Q., Liao C., Jiang G. (2021). Analysis, occurrence, toxicity and environmental health risks of synthetic phenolic antioxidants: A review. Environ. Res..

[9] Rathee P., Sehrawat R., Rathee P., Khatkar A., Akkol E.K., Khatkar S., Redhu N., Türkcanoğlu N., Sobarzo-Sánchez E. (2023). Polyphenols: Natural Preservatives with Promising Applications in Food, Cosmetics and Pharma Industries; Problems and Toxicity Associated with Synthetic Preservatives; Impact of Misleading Advertisements; Recent Trends in Preservation and Legislation. Materials.

[10] Parveen B., Rajinikanth V., Narayanan M. (2025). Natural plant antioxidants for food preservation and emerging trends in nutraceutical applications. Discov. Appl..

[11] Fadda A., Sanna D., Sakar E., Gharby S., Mulas M., Medda S., Yesilcubuk N.S., Karaca A.C., Gozukirmizi C.K., Lucarini M. (2022). Innovative and Sustainable Technologies to Enhance the Oxidative Stability of Vegetable Oils. Sustainability.

[12] Lazaridis D.G., Kitsios A.-P., Koutoulis A.S., Malisova O., Karabagias K. (2024). Fruits, Spices and Honey Phenolic Compounds: A Comprehensive Review on Their Origin, Methods of Extraction and Beneficial Health Properties. Antioxidants.

[13] Hu W., Sarengaowa, Guan Y., Feng K. (2022). Biosynthesis of Phenolic Compounds and Antioxidant Activity in Fresh-Cut Fruits and Vegetables. Front. Microbiol..

[14] Özdemir K., Demir Y. (2025). Phenolic Compounds in Exercise Physiology: Dual Role in Oxidative Stress and Recovery Adaptation. Food Sci. Nutr..

[15] Hernández-Ruiz R.G., Olivares-Ochoa X.C., Salinas-Varela Y., Guajardo-Espinoza D., Roldán-Flores L.G., Rivera-Leon E.A., López-Quintero A. (2025). Phenolic Compounds and Anthocyanins in Legumes and Their Impact on Inflammation, Oxidative Stress, and Metabolism: Comprehensive Review. Molecules.

[16] Melini V., Melini F., Luziatelli F., Ruzzi M. (2020). Functional Ingredients from Agri-Food Waste: Effect of Inclusion Thereof on Phenolic Compound Content and Bioaccessibility in Bakery Products. Antioxidants.

[17] Mikołajczak N., Tańska M., Ogrodowska D. (2021). Phenolic compounds in plant oils: A review of composition, analytical methods, and effect on oxidative stability. Trends Food Sci. Technol..

[18] https://www.statista.com/topics/4983/waste-generation-worldwide/?srsltid=AfmBOormAfcS1ny5HuBKz7qw3LcVjXKUalVzb7igFQ1lhRZjoXevN8ca.

[19] Shah A.M., Zhang H., Shahid M., Ghazal H., Shah A.R., Niaz M., Naz T., Ghimire K., Goswami N., Shi W. (2025). The Vital Roles of Agricultural Crop Residues and Agro-Industrial By-Products to Support Sustainable Livestock Productivity in Subtropical Regions. Animals.

[20] Kumar Saini R., Imtiyaj Khan M., Kumar V., Shang X., Lee J.-H., Ko E.-Y. (2025). Bioactive Compounds of Agro-Industrial By-Products: Current Trends, Recovery, and Possible Utilization. Antioxidants.

[21] Otero-Guzman N., Andrade-Pizarro R. (2025). Bioactive compounds from tropical fruit by-products: Extraction, characterization and therapeutic potential. J. Agric. Food Res..

[22] Teixeira Gomes B., Louzada Aguiar L., Tomaz Sant‘ Ana C., Vasconcelos Costa A.G., Souza Carneiro J.C., Silva P.I. (2025). Fruit By-Products: Valorization, Use and Richness of Bioactive Compounds. J. Food Process. Eng..

[23] Carvalho F., Lahlou R.A., Silva L.R. (2025). Exploring Bioactive Compounds from Fruit and Vegetable By-Products with Potential for Food and Nutraceutical Applications. Foods.

[24] https://upra.gov.co/es-co/sala-de-prensa/noticias/la-agricultura-ganaderia-caza-silvicultura-y-pesca-registro-en-2025-un.

[25] https://www.portafolio.co/economia/gobierno/colombia-podria-ser-una-despensa-mundial-de-alimentos-498747.

[26] https://www.fao.org/faostat/es/#data.

[27] Mejía G. (2024). Sustainable Solutions for FL&W Related Problems: Case Studies in Colombia.

[28] Romero-Martínez M., Andrade-Pizarro R., De Paula C. (2025). Functional compounds in tropical fruit processing by-products and intrinsic factors affecting their composition: A review. Curr. Res. Food Sci..

[29] Sánchez Sánchez J., Parada-Alfonso F., Castro-Vargas H.I. (2026). Valorization of Isabella Grape (*Vitis labrusca* L.) Pomace Through the Recovery of Nutraceuticals by Sequential Green Extraction Technologies. Foods.

[30] Castro-Vargas H.I., Baumann W., Salvador Ferreira R.S., Parada-Alfonso F. (2019). Valorization of papaya (*Carica papaya* L.) agroindustrial waste through the recovery of phenolic antioxidants by supercritical fluid extraction. J. Food Sci. Technol..

[31] Castro-Vargas H.I., Ballesteros Vivas D., Ortega Barbosa J., Morantes Medina S.J., Aristizabal Gutiérrez F., Parada-Alfonso F. (2019). Bioactive Phenolic Compounds from the Agroindustrial Waste of Colombian Mango Cultivars ‘Sugar Mango’ and ‘Tommy Atkins’—An Alternative for Their Use and Valorization. Antioxidants.

[32] Hernández-Acosta M.A., Castro-Vargas H.I., Parada-Alfonso F. (2011). Integrated Utilization of Guava (*Psidium guajava* L.): Antioxidant Activity of Phenolic Extracts Obtained from Guava Seeds with Supercritical CO_2_-Ethanol. J. Braz. Chem. Soc..

[33] (1995). General Standard for Food Additives.

[34] Chan H., Levett G. (1977). Autoxidation of methyl linoleate. Lipids.

[35] Alves de Castro L., Miranda Lizi J., Leite das Chagas E.G., Aparecida de Carvalho R., Vanin F. (2020). From Orange Juice By-Product in the Food Industry to a Functional Ingredient: Application in the Circular Economy. Foods.

[36] Nunes da Silva C., Maciel da Silva R., Lemes A.C., Dias Ribeiro F. (2024). Recovery of Phenolic Compounds by Deep Eutectic Solvents in Orange By-Products and Spent Coffee Grounds. Sustainability.

[37] Hernández-Montesinos I., Carreón-Delgado D.F., Lazo-Zamalloa O., Tapia-López L., Rosas-Morales M., Ochoa-Velasco C.E., Hernández-Carranza P., Cruz-Narváez Y., Ramírez-López C. (2024). Exploring Agro-Industrial By-Products: Phenolic Content, Antioxidant Capacity, and Phytochemical Profiling via FI-ESI-FTICR-MS Untargeted Analysis. Antioxidants.

[38] Polanía A.M., Ramírez C., Londoño L., Bolívar G., Aguilar C.N. (2023). Encapsulation of Pineapple Peel Extracts by Ionotropic Gelation Using Corn Starch, Weissella confusa Exopolysaccharide, and Sodium Alginate as Wall Materials. Foods.

[39] Mejia N.M., Castro J.P., Ocampo Y.C., Salas R.D., Delporte C.L., Franco L.A. (2020). Evaluation of antioxidant potential and total phenolic content of exotic fruits grown in Colombia. J. App. Pharm. Sci..

[40] Saini N., Anmol A., Kumar S., Wani W., Bakshi M., Dhiman Z. (2024). Exploring phenolic compounds as natural stress alleviators in plants- a comprehensive review. Physiol. Mol. Plant Pathol..

[41] Lattanzio V., Lattanzio V.T.M., Cardinali A. (2006). Role of phenolics in the resistance mechanisms of plants against fungal pathogens and insects. Phytochem. Adv. Res..

[42] Xu L., Wang X. (2025). A Comprehensive Review of Phenolic Compounds in Horticultural Plants. Int. J. Mol. Sci..

[43] Zeb A. (2020). Concept, mechanism, and applications of phenolic antioxidants in foods. J. Food Biochem..

[44] Carocho M., Ferreira I.C.F.R. (2013). A review on antioxidants, prooxidants and related controversy: Natural and synthetic compounds, screening and analysis methodologies and future perspectives. Food Chem. Toxicol..

[45] Rietjens I.M.C.M., Boersma M.G., de Haan L., Spenkelink B., Awad H.M., Cnubben N.H.H., van Zanden J.J., van der Woude H., Alink G.M., Koeman J.H. (2002). The pro-oxidant chemistry of the natural antioxidants vitamin C, vitamin E, carotenoids and flavonoids. Environ. Toxicol. Pharmacol..

[46] Gordon M.H., Pokorny J., Yanishlieva N., Gordon M. (2001). The development of oxidative rancidity in foods. Antioxidants in Food Practical Applications.

[47] Yurdunuseven Yildiz A., Öztekin S., Anaya K. (2025). Effects of plant-derived antioxidants to the oxidative stability of edible oils under thermal and storage conditions: Benefits, challenges and sustainable solutions. Food Chem..

[48] Yaragalla S., Rajendran R., Bhavitha K.B. (2025). A review on bioactive compounds and their anti-oxidative properties for the enrichment of vegetable oils. Eur. Food Res. Technol..

[49] Aydın S., Sayin U., Sezer O., Sayar S. (2021). Antioxidant efficiency of citrus peels on oxidative stability during repetitive deep-fat frying: Evaluation with EPR and conventional methods. J. Food Process Preserv..

[50] Mahungu S., Hansen S., Artz W. (1998). Volatile Compounds in Heated Oleic Acid-Esterified Propoxylated Glycerol. J. Am. Oil Chem. Soc..

[51] Onyango A.N. (2012). Small reactive carbonyl compounds as tissue lipid oxidation products; and the mechanisms of their formation thereby. Chem. Phys. Lipids.

[52] Onyango A.N., Baba N. (2010). New hypotheses on the pathways of formation of malondialdehyde and isofurans. Free Radic. Biol. Med..

[53] Omoba O., Obafaye R., Salawu S., Boligon A., Athayde M. (2015). HPLC-DAD Phenolic Characterization and Antioxidant Activities of Ripe and Unripe Sweet Orange Peels. Antioxidants.

[54] Delpino-Rius A., Eras J., Vilaró F., Cubero M.A., Balcells M., Canela-Garayoa R. (2015). Characterisation of phenolic compounds in processed fibres from the juice industry. Food Chem..

[55] Mencherini T., Campone L., Piccinelli A.L., García Mesa M., Sánchez D.M., Aquino R.P., Rastrelli L. (2013). HPLC-PDA-MS and NMR Characterization of a Hydroalcoholic Extract of *Citrus aurantium* L. var. *amara* Peel with Antiedematogenic Activity. J. Agric. Food Chem..

[56] Yahya N.A., Wahab R.A., Hamid M.A., Mahat N.A., Mohamed Huri M.A., Attan N., Hashim S.E. (2020). Statistical optimization and characterization of acoustically extracted Ananas comosus peel powder with enhanced antioxidant capacity. J. Teknol..

[57] Toledo N.M.V., Mondoni J., Harada-Padermo S.S., Vela-Paredes R.S., Berni P.R.A., Selani M.M., Canniatti-Brazaca S.G. (2019). Characterization of apple, pineapple, and melon by-products and their application in cookie formulations as an alternative to enhance the antioxidant capacity. J. Food Process Preserv..

[58] Yu Y., Wang J., Yang Y., Guo C., Li M. (2024). Influence of selected phenolic acids on advanced lipid oxidation end products generation in model systems. Food Biosci..

[59] Kristinová V., Mozuraityte R., Storrø J., Rustad T. (2009). Antioxidant Activity of Phenolic Acids in Lipid Oxidation Catalyzed by Different Prooxidants. J. Agric. Food Chem..

[60] Jasrotia S., Gupta S., Kudipady M.L., Puttaiahgowda Y.M. (2025). Advancing food preservation with quercetin-based Nanocomposites: Antimicrobial, antioxidant, and controlled-release strategies—A review. Curr. Res. Food Sci..

[61] Zhang X., Ni L., Zhu Y., Liu N., Fan D., Wang M., Zhao Y. (2021). Quercetin Inhibited the Formation of Lipid Oxidation Products in Thermally Treated Soybean Oil by Trapping Intermediates. J. Agric. Food Chem..

[62] Stojković D., Petrović J., Soković M., Glamočlija J., Kukić-Marković J., Petrović S. (2013). In situ antioxidant and antimicrobial activities of naturally occurring caffeic acid, p-coumaric acid and rutin, using food systems. J. Sci. Food Agric..

[63] Ayele G., Admassu H., Mosisa G., Desalegn A., Abeje M. (2025). Emerging techniques for catechin extraction from green tea (*Camellia sinensis*): Extraction technologies, functional potential, Toxicology, and food-industry applications: A systematic review. Cogent Food Agric..

[64] Boroski M., Giroux H.J., Visentainer J.V., Dubé P., Desjardins Y., Britten M. (2021). Tea catechin role in decreasing the oxidation of dairy beverages containing linseed oil. Int. J. Vitam. Nutr. Res..

